# The Inadequate Treatment of Pain: Collateral Damage from the War on Drugs

**DOI:** 10.1371/journal.pmed.1001153

**Published:** 2012-01-10

**Authors:** Jason W. Nickerson, Amir Attaran

**Affiliations:** 1 Institute of Population Health, University of Ottawa, Ottawa, Ontario, Canada; 2 Faculty of Law, University of Ottawa, Ottawa, Ontario, Canada; 3 Faculty of Medicine, University of Ottawa, Ottawa, Ontario, Canada

## Abstract

Jason Nickerson and Amir Attaran examine the vast inequities in medical pain relief around the world and argue that the global control of licit narcotics be shifted from the International Narcotic Control Board to WHO.

**Summary Points**

- The 1961 United Nations Single Convention on Narcotic Drugs, implemented by the International Narcotics Control Board (INCB), is still the legal foundation for international control of both licit and illicit narcotic drugs, and involves a binding system of “annual estimates” (i.e., quotas) on the amount of controlled narcotics that countries can acquire.
- In practice the Single Convention and the INCB's prohibition mandate greatly outstrips its access mandate.
- The INCB has frequently approved quotas of controlled narcotics grossly insufficient for the epidemiological prevalence of clinical pain, thus leaving millions of patients legally prohibited from accessing palliation such as morphine.
- Given the INCB's decades-long failure to administer the supply of controlled narcotics in accordance with clinical need, we propose that all legal responsibility for licit narcotics for medical and scientific purposes be shifted to the World Health Organization.

Once widely supported, the “war on drugs” has become increasingly controversial, as the political realization sinks in that it has wrought more harm than good. The Global Commission on Drug Policy, a collection of eminent former heads of state, businesspersons, and diplomats, bluntly declares that “the war on drugs has failed,” while simultaneously “[generating] widespread negative consequences for societies in producer, transit and consumer countries” [1]. The European Union's Reuter-Trautmann report witheringly finds “no evidence that the global drug problem was reduced” following the intensified criminalization of drug abuse and trafficking in the late 1990s, and that the “enforcement of drug prohibitions has caused substantial unintended harms; many [of which] were predictable” [2].

That prohibitionist drug laws often impede treating addiction or reducing its harms is already familiar to the public health community [3]–[5]. However, it is less well recognized that these same failed policies of the war on drugs inflict tremendous collateral damage on the treatment of one of the most common ailments: pain. Not just addicts, but arguably most of the world's population are victims of the failed war on drugs.

Current estimates suggest that upward of 80% of the world's population lacks access to basic pain relief [6]. Paradoxically, those 80% are mostly in poorer countries, and their need for pain relief is heightened by a relative absence of curative care such as surgery, or treatment for both communicable and non-communicable diseases causing pain (e.g., HIV/AIDS, cancer) [7]. There are many reasons for this disturbing health inequity (e.g., difficulties in procurement, lack of prescribing knowledge among health providers), but the fundamental, often overlooked reason is the cumbersome, restrictive drug laws and policies that exist at international, national, and local levels. We call the legal barriers “fundamental” because where laws forbid access to pain relief, that prohibition trumps all other reasons for the inequity.

Two treaties contain the foundation for many national drug control laws: the 1961 United Nations Single Convention on Narcotic Drugs [8], and the 1971 Convention on Psychotropic Substances [9]. Both these international laws are overseen by the International Narcotics Control Board (INCB), whose mandate is split awkwardly between promoting and controlling narcotic and psychotropic drugs and precursor chemicals [10]. On the one hand, INCB is responsible “to ensure that adequate supplies of [narcotic and psychotropic] drugs are available for medical and scientific uses,” but on the other hand, it is supposed to identify “weaknesses in national and international control systems” and to muster pressure on governments to stanch illicit uses of these same drugs. The INCB is basically in the conflicted position of both promoting and throttling the drugs it regulates.

Last year, the president of INCB admitted that the two sides of his legal mandate are out of balance: while much attention goes to prohibiting the production, supply, and use of illicit controlled substances, “equal emphasis has not been placed on the other fundamental objective of the treaties of ensuring that [licit] controlled substances are available for medical and scientific purposes” [11]. Credit must be given to INCB for recognizing this problem, but it also cannot be overlooked that the imbalance is largely the INCB's own fault. A system of “annual estimates” administered by the INCB imposes legal limits on the amount of controlled substances that countries can lawfully import. Thus, while INCB concedes that the global consumption of licit narcotics for therapeutic purposes is inadequate [12], actually its own legal regime is implicated as a cause [13].

The 2011 “Estimated World Requirements for Narcotic Drugs” [14] published by INCB provides a chilling illustration of how this institution entrenches health inequities, while ostensibly fighting illicit drugs. Under Article 21 of the Single Convention, INCB estimates are legally binding and tantamount to quotas for each controlled substance that a country may possess. These estimates are based on the country's own prediction of its pain treatment needs for the projected year, frequently using data on the number of treatments consumed in the previous year [15]. Thus, a country that consumed low amounts of drugs in previous years can become trapped in a cycle of reduced access in subsequent years, divorced from any epidemiological measure of actual clinical need.

Figure 1 presents the per capita annual entitlement of morphine in different countries relative to gross national income, based on 2011 INCB morphine annual estimates [14] and the World Bank's current data on population and gross national income [16]–[18]. The data used are available in Table S1. Figure 1 shows clearly that low-income, lower-middle-income, upper-middle-income, and high-income countries have radically different access to pain treatment under INCB annual estimates—often as much as several hundred fold different. By median, countries in these groups have a per capita entitlement to morphine of 0.502134 mg, 0.530478 mg, 3.131495 mg, and 16.876496 mg, respectively. Similar inequities exist for the other controlled narcotics.

**Figure 1:**
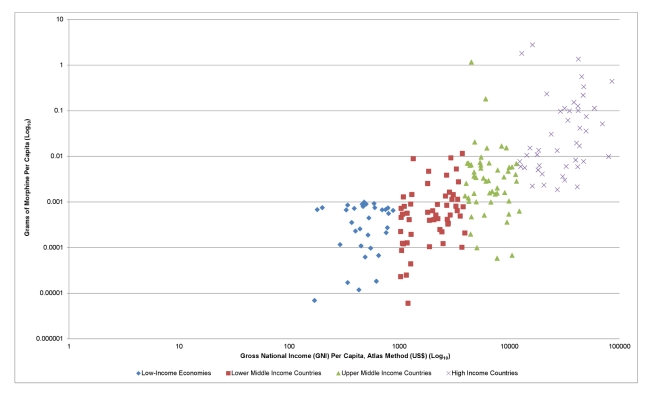
Grams of morphine per capita versus gross national income. Based on 2011 INCB morphine annual estimates [14] and World Bank data on population and gross national income [16]–[18]. Countries excluded because of incomplete data: American Samoa, Anquilla, Aruba, Ascension Island, Bermuda, British Virgin Islands, Cayman Islands, Channel Islands, Christmas Island, Cocos (Keeling) Islands, Cook Islands, Curacao, Equatorial Guinea, Falkland Islands, French Polynesia, Faeroe Islands, Gibraltar, Greenland, Guam, Isle of Man, Kuwait, Korea Dem. Rep., Kosovo, Liechtenstein, Mayotte, Monaco, Montserrat, Myanmar, Nauru, Netherlands Antilles, New Caledonia, Norfolk Island, Northern Mariana Islands, Puerto Rico, Qatar, San Marino, Saint Helena, Sint Maarten (Dutch Part), Somalia, South Sudan, St. Martin (French part), Tristan de Cunha, Turks and Caicos Islands, United Arab Emirates, Virgin Islands (US), Wallis and Fatuna Islands, West Bank and Gaza.

Common sense holds that such large per capita differences between rich and poor countries cannot correspond accurately to the epidemiological prevalence of clinical pain. We twice wrote INCB requesting it explain the methods used in deriving and ensuring the quality of its annual estimates, but received no reply.

Some argue that the INCB system of estimates should not be blamed for causing any health inequity, because the Single Convention allows countries to revise and supplement their annual estimates of controlled narcotics when needed. That argument, however, lacks evidence. If annual estimates were really so flexible, then surely in its 50-year history at least some poor countries should have arrived at estimates sufficient to meet clinical needs. Yet the data show that not even a single low-income country, not even those having generalized epidemics of HIV/AIDS and ably furnishing antiretroviral treatment, now possesses more than a derisory quota under law for furnishing pain treatment. A doctor, hospital, or aid donor who dared furnish pain treatment beyond the quota would break the law.

In short, the data clearly illustrate that lack of equity and progress in the “war on pain” is not due to a few countries lacking the infrastructure to properly use or estimate their needs for controlled narcotics, but is caused by the systemic and enduring failure of INCB to fulfill its mandate in “assisting Governments in achieving, inter alia, a balance between supply and demand” [10]. Were INCB serious about achieving balance, it would reject the ludicrously low estimates for pain medicines that it now routinely approves for poor countries—as low as 1 gram/year of morphine for a whole country in some cases [14],[19].

It is in public health emergencies, however, that INCB's estimates are the most punishing and deplorable for health. The chronic shortage of analgesics in Haiti—whose population of 9.7 million people is allocated only 671 grams of morphine, 117 grams of codeine, 83 grams of fentanyl, and 309 grams of pethidine—became acutely intolerable after the January 2010 earthquake forced amputations to be performed without anesthesia or post-operative analgesia [20]. To address this, a full week after the earthquake, the INCB issued a diplomatic note advising countries that narcotics exports to Haiti could proceed “even in the absence of…import authorizations” normally required by the Single Convention [21]. But INCB's legal interpretation was flagrantly wrong: under Article 21(4)(b)(ii) of the Single Convention, which governs emergencies, import authorizations continue to be required as normal pursuant to Article 31(5). With Haiti's government interred under the rubble, obviously no import authorizations could be forthcoming.

The Haiti earthquake, in other words, put INCB in the clumsy position of urging governments to unlawfully violate the Single Convention's rigid rules for import authorizations—something governments may or may not be willing to do, but surely an argument that the Single Convention is ill-considered, dangerous to public health, and overdue for amendment.

Regrettably, many national governments appear to have followed INCB's example of not balancing narcotic drugs with the demands of public health [22]. Governments may restrict the types of narcotics that are available, the route by which they can be administered, or the setting in which they can be delivered. In Iran, for example, morphine is not a registered medicine, and tablets are unavailable in the regular market, which all but eliminates the possibility of receiving adequate analgesia in the community rather than the hospital [23]. In Jordan, a prescription for morphine is valid for only ten days for cancer patients, and three days for other patients, placing an impractical burden on patients and the health care system to renew prescriptions constantly [23],[24].

It is now timely to rebalance drug policy, so that the requirements of pain patients for licit narcotics are met [25]. Pain has to be viewed not just as a clinical problem in need of better treatment modalities, but as a social problem in need of wiser international and national policies, laws, and institutions. The Single Convention is 50 years old, so there can be no argument that reassessing it is premature, or that global circumstances have not changed. At the time it was written (in 1961) tertiary care and pain control was commonplace in only a handful of rich countries, and none of the countries that are today recognized as “emerging” (e.g., Brazil, China, India, Indonesia, South Africa). With the demographic transition now underway in these countries and aging populations worldwide, it is all but certain that the incidence of non-communicable diseases that require opioid analgesics during treatment or palliative care shall increase. The health inequities produced by the Single Convention's system of estimates are therefore poised to worsen, unless there is a corrective intervention.

Regrettably, INCB has already tried, and failed, to initiate reform from within. Nearly two decades ago (in 1994), INCB carried out an assessment of the effectiveness of its treaty regime [26] in which it noted that the “[treaty objective] of ensuring an adequate supply of narcotic drugs, especially opiates used for medical purposes, has not been universally achieved” and that little of the world's morphine (20%) was consumed in developing countries. Yet strikingly, INCB concluded from this evidence that it did “not appear necessary to substantially amend the international drug control treaties,” and instead, INCB merely exhorted countries “never [to] hinder the availability of drugs for legitimate medical purposes.”

Fifteen years later, INCB's considered decision to choose exhortation over treaty reform has proved resoundingly unsuccessful, and the inequitable, inadequate supply of controlled narcotics for pain control persists. With INCB having botched that corrective opportunity so badly, it would be naïve to entrust INCB with spearheading other corrective interventions in the future. The need to relocate the legal framework and responsibilities to a different institution is irrefutable.

We propose amending the Single Convention so as to transfer the part of INCB's mandate and funding that deals with licit controlled medicines to the World Health Organization (WHO). Doing so would more closely integrate that public health function with WHO's existing efforts to improve access to essential medicines, rational prescribing, and health system strengthening [27]–[29]. There is no doubt that the mandate fits within WHO's legal competences, for WHO's constitution gives it the authority “to perform such duties as may be assigned” by treaty and “to act as the directing and co-ordinating authority on international health work” [30]. Transferring the public health responsibility for controlled medicines from INCB to WHO would end the impossibly contradictory situation in which INCB is mandated both to restrict and to promote access to controlled medicines, while also putting an agency with competence and commitment to health equity in charge. Certainly WHO is the better agency to accurately estimate the epidemiological need for pain control and to coordinate emergency interventions such as Haiti's.

In conclusion, the war on pain, much like the war on drugs which eclipses it, is a failure, and a strict prohibition mind-set has served neither. Five decades after the Single Convention, and two decades after the INCB initiated its last ineffective attempt at reforms, it would be exceedingly naïve not to conclude that this experiment has run its course. Attention must now shift to creating better legal frameworks that extricate pain treatment from drug prohibition, and that formally transfer some responsibilities and funding from INCB to WHO, so that health equity plays a part in narcotics control policy. To reject this conclusion is to continue embracing a cruel system in which persons needlessly lack treatment for pain, for the stubborn pursuit of narcotics prohibition, which others have found no longer desirable.

## Supporting Information

Table S1 **Morphine grams per capita versus gross national income.**
(XLS)

## Abbreviations

INCB: International Narcotics Control Board
WHO: World Health Organization

## References

[1] Global Commission on Drug Policy (2011). Report of the Global Commission on Drug Policy.. http://www.globalcommissionondrugs.org/Report.

[2] Reuter P, Trautmann F (2009). A report on global illicit drugs markets 1998–2007.. http://ec.europa.eu/justice/anti-drugs/files/report-drug-markets-short_en.pdf.

[3] Wood E, Werb D, Marshall BDL, Montaner JSG, Kerr T (2009). The war on drugs: a devastating public-policy disaster.. Lancet.

[4] Wood E, Werb D, Kazatchkine M, Kerr T, Hankins C (2010). Vienna Declaration: a call for evidence-based drug policies.. Lancet.

[5] International Centre for Science in Drug Policy (2010). The Vienna declaration.. http://www.viennadeclaration.com/the-declaration/.

[6] Scholten W, Nygren-Krug H, Zucker HA (2007). The World Health Organization paves the way for action to free people from the shackles of pain.. Anesth Analg.

[7] Taylor AL (2007). Addressing the global tragedy of needless pain: rethinking the United Nations single convention on narcotic drugs.. J Law Med Ethics.

[8] United Nations (1961). Single convention on narcotic drugs.. http://www.unodc.org/pdf/convention_1961_en.pdf.

[9] United Nations (1971). Convention on psychotropic substances.. http://www.incb.org/pdf/e/conv/convention_1971_en.pdf.

[10] International Narcotics Control Board (2011). Mandate and functions.. http://www.incb.org/incb/mandate.html.

[11] World Health Organization (2011). Ensuring balance in national policies on controlled substances: guidance for the availability and accessibility of controlled medicines.. http://www.who.int/medicines/areas/quality_safety/GLs_Ens_Balance_NOCP_Col_EN_sanend.pdf.

[12] International Narcotics Control Board (2011). Availability of internationally controlled drugs: ensuring adequate access for medical and scientific purposes.. http://www.incb.org/pdf/annual-report/2010/en/supp/AR10_Supp_E.pdf.

[13] International Narcotics Control Board (2010). Report of the International Narcotics Control Board for 2010.. http://www.incb.org/pdf/annual-report/2010/en/AR_2010_English.pdf.

[14] International Narcotics Control Board (2011). Estimated world requirements of narcotic drugs in grams for 2011 (May update).. http://www.incb.org/pdf/e/estim/2011/EstMay11.pdf.

[15] De Lima L (2004). Opioid availability in Latin America as a global problem: a new strategy with regional and national effects.. J Palliat Med.

[16] World Bank (2011). Population, total.. http://data.worldbank.org/indicator/SP.POP.TOTL.

[17] World Bank (2011). GNI per capita, Atlas method (current US$).. http://data.worldbank.org/indicator/NY.GNP.PCAP.CD.

[18] World Bank (2011). Country and lending groups.. http://data.worldbank.org/about/country-classifications/country-and-lending-groups.

[19] Lohman D, Schleifer R, Amon J (2010). Access to pain treatment as a human right.. BMC Med.

[20] Missair A, Gebhard R, Pierre E, Cooper L, Lubarsky D (2010). Surgery under extreme conditions in the aftermath of the 2010 Haiti earthquake: the importance of regional anesthesia.. Prehosp Disaster Med.

[21] International Narcotics Control Board (2010). Ref.: E/INCB/NAR/C.L. 05/2010.. http://www.incb.org/documents/For_Highlights_box/INCB_Circular_letter_to_governments_Haiti_200110.pdf.

[22] MacDonald DM, Finley GA (2001). Governmental barriers to opioid availability in developing countries.. J Pharmaceutical Care Pain Symptom Control.

[23] Human Rights Watch (2011). Global state of pain treatment: access to medicines and palliative care.. http://www.hrw.org/sites/default/files/reports/hhr0511W.pdf.

[24] Stjernsward J, Ferris FD, Khleif SN, Jamous W, Treish IM (2007). Jordan Palliative Care Initiative: a WHO demonstration project.. J Pain Symptom Manage.

[25] Liberman J, O'Brien M, Hall W, Hill D (2010). Ending inequities in access to effective pain relief?. Lancet.

[26] International Narcotics Control Board (1994). Evaluation of the effectiveness of the international drug control treaties, Part 3.. http://www.incb.org/pdf/e/ar/incb_report_1994_supplement_en_3.pdf.

[27] Laing R, Hogerzeil H, Ross-Degnan D (2001). Ten recommendations to improve use of medicines in developing countries.. Health Policy Plan.

[28] Travis P, Bennett S, Haines A, Pang T, Bhutta Z (2004). Overcoming health-systems constraints to achieve the Millennium Development Goals.. Lancet.

[29] World Health Organization (2011). WHO's pain ladder.. http://www.who.int/cancer/palliative/painladder/en.

[30] World Health Organization (2006). Constitution of the World Health Organization, 45th edition.. http://www.who.int/governance/eb/who_constitution_en.pdf.

